# Comparing antibiotic self-medication in two socio-economic groups in Guatemala City: a descriptive cross-sectional study

**DOI:** 10.1186/s40360-015-0011-3

**Published:** 2015-04-27

**Authors:** Brooke M Ramay, Paola Lambour, Alejandro Cerón

**Affiliations:** Department of Pharmaceutical Chemistry, Universidad del Valle de Guatemala, Guatemala City, Guatemala; Department of Anthropology, University of Denver, Denver, Colorado USA

**Keywords:** Self-medication, Guatemala, Antibiotic use, Socio-economic factors, Role of the pharmacist

## Abstract

**Background:**

Self-medication with antibiotics may result in antimicrobial resistance and its high prevalence is of particular concern in Low to Middle Income Countries (LMIC) like Guatemala. A better understanding of self-medication with antibiotics may represent an opportunity to develop interventions guiding the rational use of antibiotics. We aimed to compare the magnitude of antibiotic self-medication and the characteristics of those who self-medicate in two pharmacies serving disparate socio-economic communities in Guatemala City.

**Methods:**

We conducted a descriptive, cross-sectional study in one Suburban pharmacy and one City Center pharmacy in Guatemala City. We used a questionnaire to gather information about frequency of self-medication, income and education of those who self-medicate. We compared proportions between the two pharmacies, using two-sample z-test as appropriate.

**Results:**

Four hundred and eighteen respondents completed the survey (221 in the Suburban pharmacy and 197 in the City Center pharmacy). Most respondents in both pharmacies were female (70%). The reported monthly income in the suburban pharmacy was between $1,250.00-$2,500.00, the city-center pharmacy reported a monthly income between $125.00- $625.00 (p < 0.01). Twenty three percent of Suburban pharmacy respondents and 3% in the City Center pharmacy completed high school (p < 0.01). Proportion of self-medication was 79% in the Suburban pharmacy and 77% in City Center pharmacy. In both settings, amoxicillin was reported as the antibiotic most commonly used.

**Conclusions:**

High proportions of self-medication with antibiotics were reported in two pharmacies serving disparate socio-economic groups in Guatemala City. Additionally, self-medicating respondents were most often women and most commonly self-medicated with amoxicillin. Our findings support future public health interventions centered on the regulation of antibiotic sales and on the potential role of the pharmacist in guiding prescription with antibiotics in Guatemala.

**Electronic supplementary material:**

The online version of this article (doi:10.1186/s40360-015-0011-3) contains supplementary material, which is available to authorized users.

## Background

Self-medication with antibiotics occurs worldwide, fostering antibiotic misuse and antimicrobial resistance. The World Health Organization (WHO) recently reported alarming levels of resistance to penicillin, fluoroquinolones and third generation cephalosporins in member countries [1]. The misuse of antibiotics poses a serious risk to infectious disease control and public health in general [1,2]. In Low to Middle Income Countries (LMIC) like Guatemala antibiotics are sold essentially as over-the-counter medications. In addition to easy access of antibiotics, self-medication is highly prevalent in LMIC and Organization for Economic Co-operation and Development (OECD) countries although no patterns in the characteristics of those who self-medicate have been established [3,4]. What is clear, is that patients practicing self-medication in LMIC are often unaware of potential problems that may arise [5] including side effects, antimicrobial resistance, or worsening of symptoms. [6-9] Additionally, self-medicating individuals don’t have pertinent information regarding medications’ side effects and dosing instructions [10,11]. These problems are to be expected in self-medicating patients given that patients often obtain medication advice from non-professionals [3], family and friends [12]. In these cases self-medication may lead to irrational use, poor adherence to regimens, side-effects and overuse of antibiotics.

Evidence shows excessive self-medication practice in Latin America [6,7,13,14] but data indicating types of drugs and factors associated with self-medication is limited and often contradictory. In Argentina, Brazil, Chile, Colombia, Costa Rica, and Nicaragua, authors attributed the high prevalence of self-medication to poor access to health care services [13]. In Peru there were no significant differences in self-medication practices associated with gender, occupation, educational level or being head of the household [14]. In Honduras, higher proportions of self-medicating patients have been reported among those living in urban areas, but socio-economic status was not associated with self-medication [6].

Evidence regarding self-medication and its relationship to educational level and socio-economic status is mixed. In Peru, authors found that education had no significant effect on self-medication [14], while two studies in Sudan and one in Jordan found that self-medication was associated with higher literacy levels [15-17]. In one European study, higher educational level predicted higher self-medication rates [4]. Educational level does not clearly predict proportions of those who self-medicate, the same is true for socio-economic status where data is inconsistent among groups. In Jordan, one study showed positive association between self-medication and low income [17]; in Syria, patients with a middle income more frequently self-medicated [8], whereas in the United Arab Emirates there was no association to income but rather self-medication was related to ethnicity [18].

In addition to the varying data surrounding characteristics of those who self-medicate, the official health system has been ambiguous and contradictory about self-medication, recommending it in some cases while challenging it in others [19]. Multiple factors facilitate high prevalence of self-medication, such as poor access to health care providers, low quality of health services, high costs of medications, absence of regulations regarding the promotion and sale of medications, easy access in pharmacy outlets without a prescription, and publicity of pharmacy chains [20].

In Latin America pharmacists have shown interest in participating in patient-care to aid in safe medication practice [21], which may be more effective in curbing antibiotic misuse when the characteristics of self-medication with antibiotics are better understood. In order to design these types of educational interventions in the community it is important to understand the environment surrounding the practice of self-medication: motives for self-medicating, ways of self-medicating, and characteristics of those who self-medicate.

In this study we aimed to compare the magnitude of antibiotic self-medication and the characteristics of those who self-medicate with antibiotics in two pharmacies serving disparate socio-economic communities in Guatemala City.

## Methods

From May to August of 2013 we carried out a descriptive cross-sectional study in two pharmacies in Guatemala City. We used purposeful sampling to select two private pharmacies serving different segments of the population according to key informants from pharmacy staff members. The first pharmacy was located in San Cristobal (zone 8 of Mixco, according to the local nomenclature), a suburb that is part of the city’s metropolitan area serving clients characterized as professional or executive employees with higher levels of education and higher purchasing power. The second pharmacy, located in historical City Center (zone 1, using the local nomenclature), serves clients generally characterized as being working class and with lower levels of education and lower purchasing power.

Sample size was calculated for each pharmacy using Epidat 4.0 based on a population of 350 patients arriving to the pharmacy weekly, assuming that 50% of the population self-medicates, a precision of 5% and a 95% confidence level. Customers who purchased antibiotics without a prescription were invited to participate in the study. Written informed consent was obtained and participants were given a brief verbal definition of the practice of self-medication and the opportunity to ask any questions regarding the study or self-medication with antibiotics. Participants were asked to complete the questionnaire, which consisted of a brief introduction of study objectives and a definition of self-medication: “Self-medication occurs when patients obtain and use medications without a prescription from a doctor, meaning that patients make a personal decision to seek treatment for their illness.” We determined which antibiotics were being used for self-medication by documenting the generic name of the antibiotic at the time the participant purchased the medication. Potential participants were excluded from the study if they were less than 16 years old, had seen a doctor that day, were already taking antibiotics, belonged to a vulnerable population (HIV positive, elderly or underage), were under the influence of alcohol or drugs, and/or did not understand Spanish.

Data was collected by a questionnaire [1] that was designed based on instruments used in previous studies [10,22,23]. The questionnaire was validated by interviewing approximately 20 customers with the aim of detecting comprehension problems and to assess if the questions responded to the research aims. The instrument consisted of 22 questions: 21 multiple choice and 1 open ended question. Multiple responses were allowed for the following items: 1) Respondent symptoms provoking self-medication, 2) Reasons for self-medicating, and 3) Locations where respondents purchased antibiotics for self-medicating. We gathered information about frequency of self-medication, symptoms that provoked self-medication, with which antibiotics patients self-medicated, whom they went to for advice upon self-medicating, and if they read the antibiotic information handout. We defined antibiotics as the following: medications that treat bacterial and protozoal infections, and that are found on the World Health Organization’s (WHO) Model list of Essential medicines and on the Guatemalan national “basic list” of medications [24,25]. The questionnaire was administered in a private area of the pharmacy from May to August of 2013, Monday through Friday between the hours of 9 am and 4 pm (until the target number of questionnaires were obtained). Participants were given the option of self-administering the questionnaire or having the researcher register their responses through verbal response.

Data analysis compared proportions of self-medication between the two pharmacies, using two-sample z-test as appropriate. The Research Ethics Committee at the Universidad del Valle de Guatemala approved all research materials before the study began.

## Results

### Socio-demographic characteristics

A total of 418 people responded to the survey, 221 in the Suburban pharmacy and 197 in the City Center pharmacy, with approximately 70% of participants self-administering the questionnaire. The majority of participants in the Suburban pharmacy and City Center pharmacy were female (70%) and between the ages of 20-29 years old (28%, 39% respectively).

Although the majority of study participants were salaried employees (62% Suburban pharmacy, 55% City Center), there was a marked difference between pharmacies when looking at the monthly income and educational level. In the Suburban pharmacy the monthly income of participants was between $1,334.00-$2,666.00, whereas in the City-Center pharmacy the monthly income was between $0.00- $667.00 (p < 0.01). The proportion of respondents who had completed a high school education was 27% in the Suburban pharmacy and 3% in the City Center pharmacy (p < 0.01). The demographic characteristics of the respondents are shown in Table 1.

**Table 1:** **Demographics of self-medication survey respondents visiting two pharmacies in the Guatemala City area**

**Basic demographics**	**# Suburb n = 221, (%)**	**# City center n = 197, (%)**
Age		
16-19	14 (6%)	15 (7%)
20-29	63 (29%)	77 (39%)
30-39	59 (27%)	45(23%)
40-49	43 (19%)	25 (13%)
50 and above	42 (19%)	35 (18%)
**Gender**		
Female	155 (70%)	137 (70%)
Male	66 (30%)	60 (30%)
**Marital Status**		
Married	124 (56%)	88 (45%)
Single	28 (13%)*	53 (27%)*
Other	69 (31%)	56 (28%)
**Educational level**		
Less than Middle School	60 (27%)*	131 (66%)*
Middle School Education	55 (25%)	52 (26%)
High School Education	59 (27%)*	6 (3%)*
College Education	47 (21%)*	8 (4%)*
**Income**		
$0-$667	95 (43%)*	153 (77%)*
$668-$1,333	38 (17%)*	9 (5%)*
$1,334-$2,666	88 (40%)*	35 (18%)*
**Occupation**		
House wife	45 (20%)	49 (25%)
Salaried employee	138 (62%)	108 (55%)
Independent worker	22 (10%)	17 (8%)
Other	16 (7%)	23 (12%)

*Significant difference between suburb and city center pharmacies, p < 0.01.

### Magnitude of self-medication

The proportion of self-medication with antibiotics was high in both pharmacies: 79% in the Suburban pharmacy and 77% in City Center pharmacy. The two primary reasons for self-medicating in both pharmacies were time constraints for doctors’ visits (38% Suburb, 56% City Center p < 0.01) and purchasing convenience (27% Suburb, 17% City Center). Frequency of self-medication differed between pharmacies. Suburban pharmacy participants reported self-medicating once a month (35%) or once a year (30%). In contrast City Center pharmacy respondents reported self-medicating once a week (33%) or once a year (34%). Although many respondents claimed to buy medications several times a year, most reported seeing a doctor only once a year (City Center 48%, Suburban pharmacy 51%).

In both settings, amoxicillin was most commonly purchased for self-medication, followed by tetracycline and sulfamethoxazole/trimethoprim, as it is detailed in Table 2. Flu-like symptoms were the most common reason for self-medicating in the Suburban and City Center pharmacy (33%, 32%, respectively), followed by fever and pain as shown in Table 3.

**Table 2:** **Number of respondents purchasing antibiotics when self medicating**

**Antibiotic purchased for use in self-medication**	**Suburb (n = 221)**		**City center (n = 197)**	
	**Number of respondents**	**%**	**Number of respondents**	**%**
Amoxicillin	114	51.58	82	41.62
Tetracycline	34	15.38	55	27.92
Trimethoprim-sulfamethoxazol	13	5.88	20	10.15
Erithromycin	11	4.98	18	9.14
Ciprofloxacin	9	4.07	9	4.57
Cefadroxil	0	0.00	4	2.03
Cefixime	0	0.00	4	2.03
Amoxicilin/Clavulanic Acid	9	4.07	3	1.52
Azithromycin	5	2.26	2	1.02
Secnidazol	8	3.62	0	0.00
Albendazol	6	2.71	0	0.00
Metronidazol	6	2.71	0	0.00
Levofloxacin	3	1.36	0	0.00
Ceftriaxone	2	0.90	0	0.00
Clarithromycin	1	0.45	0	0.00
Total	221	100	197	100

**Table 3:** **Characteristics of self-medication in two Guatemala City pharmacies**

	**Number of respondents in Suburb (n = 221)**	**Number of respondents in City Center (n = 197)**
**Symptoms resulting in self-medication** ^**+**^		
Cold/flu	74 (33%)	65 (33%)
Pain	66 (30%)	54 (27%)
Fever	39 (18%)	41 (21%)
Stomach ache	22 (10%)	26 (13%)
Diarrhea	10 (5%)	5 (3%)
Allergy	10 (4%)	6 (3%)
**Reasons for self-medicating** ^**+**^		
Lack of time and to save time	84 (38%)*	110 (56%)*
Easily purchasable medications from pharmacies	60 (27%)	34 (17%)
Economic reasons (High costs of visits to doctor/Low cost of purchasing drugs)	34 (15%)	29 (15%)
Simple sign and symptom of a disease	20 (9%)	18 (9%)
Convenient (ease of curing perceived symptoms)	18 (8%)	3 (2%)
Lack of trust toward doctors	4 (2%)	3 (2%)
**Locations for obtaining medications** ^**+**^		
Pharmacies	172 (77%)	139 (70%)
Supermarket	20 (9%)*	0 (0%)*
Corner stores	19 (8%)*	58 (29%)*
From home (previously purchased)	9 (4%)*	0*
**Frequency of self-medication**		
One time per week	42(19%)*	65(33%)*
One time per month	77(35%)*	12(6%)*
Two times per month	1(0.5%)	6(3%)
Every two months	1(0.5%)	2(1%)
Every three months	5(2%)	8(4%)
Every six months	16(7%)	21(11%)
Two times per year	2(1%)	0
One time per year	66(30%)	67(34%)
Never	11(5%)	16 (8%)
***Regarding the antibiotic information handout***		
Do not read antibiotic information handout	176 (80%)*	188 (95%)*
Read antibiotic information handout	45 (20%)*	9 (5%)*
**How it can effect one's health, 1 negative effect 10 positive effect**		
1 on a scale of 10	0 (0%)*	0 (0%)*
2-4 on a scale of 10	37 (17%)	24 (12%)
5 on a scale of 10	45 (20%)	35 (18%)
6 on a scale of 10	67 (30%)	40 (20%)
7 on a scale of 10	42 (19%)	46 (23%)
8-10 on a scale of 10	30 (14%)	44 (22%)
**Who respondents go to for advice**		
Pharmacy employee	84 (38%)*	8 (4%)*
Family	81 (37%)*	129 (65%)*
Friend	52 (23%)	60 (30%)
Other	4 (2%)	0 (0%)

*Significant difference between suburb and city center pharmacies, p < 0.01.

^**+**^Multiple responses were allowed for these items.

### Characteristics of self-medication

In the Suburban pharmacy, respondents purchased self-medicating products in pharmacies (77%) and supermarkets (9%). In the City Center pharmacy, the majority of respondents purchased antibiotics in pharmacies (70%) followed by neighborhood stores (or “*tiendas*” in Spanish) (29%).

Participants were asked to rate, on a scale of 1-10, the effect self-medication could have on one’s health (1 being a negative effect and 10 being a positive effect). The majority of respondents, 63% of Suburban and 65% of City Center participants, responded marking 6 and above, perceiving little or no negative effect in self-medication (see Table 3).

Participants from both pharmacies obtained information regarding self-medication with antibiotics through advice rather than by reading patient handouts. In the Suburban pharmacy, respondents sought advice from pharmacy technicians (38%), followed by family (36%) and friends (23%). In contrast, City Center respondents spoke with family members (65%) or friends (30%) while only 4% went to pharmacy employees for advice. A large majority of respondents in both the Suburban and City Center pharmacies indicated that they did not read the instructions or the patient handout accompanying the antibiotic before self-medicating (80% and 95%, respectively). These findings are summarized in Table 3.

## Discussion

High proportions of self-medication were similar in both pharmacies, despite the differences in monthly income and educational level. This differs with findings in other studies. One comparative study in Brazil documented a higher prevalence of self-medication in higher socio-economic classes versus lower socio-economic classes; higher socio-economic patients paid out of pocket for their medications and lower socio-economic patients had free access to medication. In this Brazilian study, paying for medications was a positive factor associated to self-medication [26]. Another study in Mexico showed that low socio-economic status and lower educational level were positively associated to self-medication [11]. Practices in self-medication and their relation to socio-economic status have been defined in these settings, but to our knowledge this has not been previously established in Guatemala. Our findings suggest that self-medication with antibiotics in this urban Guatemala City setting is high despite differences in monthly income and educational level.

More women came to pharmacies to self-medicate with antibiotics than men in both settings. This is similar to a recent study carried out in Chile whose findings indicated that 73% of those who self-medicated were female [10]. The high proportion of females who self-medicate has also been reported in several LMIC, OECD and European countries [3] with the exception of Nepal, Syria and the United Arab Emirates [8,18,27]. In Mexico women have been reported to self-medicate themselves or their children more often than men, in this context, authors agreed that women should be targeted in health-education campaigns [7]. One study in rural Peru interviewed heads of the household in order to understand characteristics surrounding those who self-medicate. The head of the household was predominately male and responded more frequently to the questionnaire, however, there was no significant association found between gender and self-medicating practices [14]. The patterns of women who self-medicate are unknown in rural areas of Guatemala, in other urban areas outside of Guatemala City, and in areas with high proportions of indigenous people who do not use Spanish as their first language. These factors may affect the proportion of men and women who obtain medications from the pharmacy in order to self-medicate. Further investigation regarding gender and self-medication is warranted in Guatemala given that the gender of those who self-medicate may vary based on pharmacy and socio-cultural practices of each region.

We found that participants of a lower socio-economic status go to family or friends for advice when self-medicating, whereas those of a higher socio-economic status more frequently talk to a pharmacy technician although they also rely on family. In a recent review of 70 studies looking at self-medication, 8 studies cited family as the major source of information for those who self-medicate. Seven studies cited friends and only 6 studies recorded pharmacists as the primary source of drug-information [3]. A recent study in older Mexican participants showed that respondents seek out advice first from family (30%), followed by a pharmacist (27%) [11]. In Guatemala we see a significant difference in whom participants are willing to approach for advice based on their socio-economic status. These findings would likely be important when designing educational programs aiding participants in the selection of self-medication.

The majority of respondents in both pharmacies indicated that self-medication has a positive effect on their health. Previous studies have emphasized the dangers in self-medicating with antibiotics. Side effects, incorrect drugs or dosages and antibiotic resistances are all factors that make the practice problematic [1,7,28]. Patient awareness surrounding the “how”, “why” and “when” to use antibiotics as well as the risks involved in self-medication with antibiotics may be created through educational initiatives. One study reviewed LMIC pharmacy interventions and placed an emphasis on the educational services pharmacists may provide in order to improve outcomes [9]. These types of services must go beyond classifying medication use as “good or bad” [29]. If educational services are to be implemented, they must be all-inclusive resulting in a comprehensive educational plan for those who self-medicate [6,9,13]. There must be support within the local health-system giving incentive to form sustainable educational programs in the community. The gender of those who self medicate, how the socio-economic status influences self-medication, with whom respondents are willing to go to for advice, and techniques by which participants receive information about self-medicating all contribute to developing educational and political movements to ensure the safety and efficacy of antibiotic use.

Participants in this study did not read the antibiotic information handout associated with the medications they purchased, regardless of their socio-economic status and educational level. Educational level plays an important role when deciding how to effectively design drug information for participants [9]. Informing patients about a medication’s indications, posology and side effects using means other than patient handouts that accompany medications is an important challenge if antibiotic use is to be addressed. Although all participants had some level of schooling, patient handouts accompanying medications may be inadequate in Guatemala given that the majority of self-medicators did not have a secondary school education. Combining the risks of self-medication with the demographic data we have gathered, we provide a basis for initiating educational policies surrounding medication use in the urban setting of Guatemala.

The majority of respondents purchased antibiotics from pharmacies where antibiotics are sold in the absence of any medical regulation, contributing to irrational use and antimicrobial resistance. In most LMIC, the debate of antibiotic regulation skews towards authorization of unregulated vendors selling antibiotics in order to maintain reasonable access to medications [30,31]. Additionally, competing interests of the pharmaceutical industry and pharmacy chains promote unregulated use of antibiotics [30]. Nevertheless, restricted and regulated use of antibiotics is of public health concern both locally and for many globally recognized organizations [2,28].

There is currently no law in Guatemala requiring the continual presence of a pharmacist in the pharmacy. Trained health care professionals do not monitor the sale and dispensing of antibiotics in Guatemala in community pharmacies, and there is no law requiring a prescription for antibiotic use; prescriptions are only required for controlled substances. Both factors -absence of health care professionals in the pharmacy and lack of regulations- lead to irrational use of antibiotics and antimicrobial resistance. This presents an opportunity for developing the role of the pharmacist in guiding the rational use of antibiotics in Guatemala where we have shown that a proportion of respondents seek advice from pharmacists. Fundamental to the policy development surrounding the role of a pharmacist is the establishment of associated laws regulating the dispensing of medications [14]. Relationships with other health care professionals, social pressures and conflicts of business and professional roles must be taken into account in this type of policy development [9]. Restructuring and eventual development of the role of the pharmacist may improve safe and rational use, affordability and accessibility of antibiotics in Guatemala [9,12].

Limitations of this study include those inherent to the cross-sectional research design, as well as the use of purposeful sampling for selecting the pharmacies. The study is not population-based and therefore its results cannot be assumed to be generalizable to all pharmacies in Guatemala City or to Guatemala City. The study did not ask qualitative questions that allowed participants to provide their own explanations and meanings around self-medication.

The results of our study contribute to a better understanding of why people self-medicate, the characteristics of those who self-medicate and how people self-medicate in Guatemala and should be complemented with further investigations that include pharmacies located in other urban and rural settings. It is also important to determine which, if any, side effects participants may experience as a result of self-medicating with antibiotics and if their perceived risk of self-medication changes across rural versus urban settings. Also, it may be important to know if participants perceive self-medication as “curative” or, if as a result of their practice, they have to see a physician to improve health outcomes. Additionally, further studies may focus on health literacy and the health systems dimensions of this problem.

## Conclusions

The high proportion and factors contributing to self-medication with antibiotics in Guatemala City are similar in two disparate socio-economic pharmacies. In this setting, women come to the pharmacy more often than men in order to self-medicate and perceive little risk in its practice. Those of higher socio-economic status in Guatemala City are willing to speak to pharmacy personnel for advice regarding self-medication and, although future studies are necessary, this study sets the stage for future policy development regarding the role of the pharmacist in addressing self-medication with antibiotics. This type of role, however, may have a limited public health impact if there are no changes in the regulation of antibiotic promotion, sale and use in Guatemala.

## Additional file

Additional file 1:
**Antibiotic self-medication questionnaire_Spanish.**
