# Supplementary material for: Comparing antibiotic self-medication in two socio-economic groups in Guatemala City: a descriptive cross-sectional study
Source: BMC Pharmacol Toxicol. 2015 Apr 27;16:11. doi: 10.1186/s40360-015-0011-3 (PMC4418049; doi:10.1186/s40360-015-0011-3)
Supplement: Additional file 1: — Antibiotic self-medication questionnaire_Spanish. [file 40360_2015_11_MOESM1_ESM.docx]

Código del participante. _________

Universidad del Valle Guatemala

Departamento de Química Farmacéutica

CUANTIFICACIÓN DE AUTOMEDICACIÓN DE ANTIBIÓTICOS EN DOS

FARMACIAS COMUNITARIAS PRIVADAS, UBICADAS EN MIXCO Y LA CIUDAD DE GUATEMALA

La **automedicación** ocurre cuando los pacientes consiguen y utilizan los medicamentos sin receta médica, es decir, deciden en forma personal buscar tratamiento para su enfermedad.

Fecha: ______

1. Edad: _____
2. Sexo: □ F □ M

1. Dirección donde vive: _____________________
2. Estado civil:

□ Casado □ Soltero □ Otro

1. Ocupación:

□ Empleado □ Ama de casa □ Trabajador Independiente

□ Otra

1. Estudios:

Educación Básica: □ Completa □ Incompleta

Educación Media: □ Completa □ Incompleta

Educación Superior: □ Completa □ Incompleta

1. Ingreso mensual (Quetzales):

□ Q 0.00-5.000

□ Q 5.001-10.000 □ Q 10.000-20.000

1. Alguna vez; ¿Ha tomado medicamentos sin receta de un doctor?

□ SI

□ NO

1. ¿Ha tomado medicamentos sin receta de un doctor en el último AÑO?

□ SI □ NO

1. ¿Ha tomado medicamentos sin receta de un doctor en el último MES?

□ SI □ NO

1. ¿Ha tomado medicamentos sin receta de un doctor en la última SEMANA?

□ SI □ NO

1. ¿Con qué frecuencia toma medicamentos sin receta de un doctor?

□ 1 vez por semana

□ 1 vez al mes

□ Otro______

1. ¿Con qué frecuencia asiste a consulta con un doctor?

Año ________

Mes ________

Semana _______

1. ¿Cuál/es son los principales síntomas por lo que solicita/toma antibiótico sin receta?

□ Alergias □ Ardor/dolor estomacal □ Diarrea

□ Dolores □ Fiebre □ Gripe

1. ¿Podría dar el nombre y frecuencia de los antibióticos que comúnmente toma y los ha obtenido sin RECETA de un doctor?

| Medicamentos | ¿En el último año? | En el último mes? | En la última semana? |
| --- | --- | --- | --- |
|  |  |  |  |
|  |  |  |  |
|  |  |  |  |
|  |  |  |  |

1. Habitualmente cuando toma un antibiótico, Lee las indicaciones?

□ SI □ NO □ No sabe lo qué es.

1. ¿Alguna persona le ha recomendado antibióticos sin receta de un doctor?

□ SI (Continúe la encuesta) □ NO (no responde esta pregunta)

1. ¿Quién/enes?

□ Amigos, vecinos □ Consejo Familiar □ Dependiente de Farmacia

□ Otro _____________________________________

1. ¿Qué lo motiva a usar antibióticos sin receta?

□ Considera tener conocimientos

□ Enfermedad leve (No es necesario consultar a un doctor)

□ Fácil acceso para comprar sin receta

□ Falta de tiempo para ir al médico

□ Motivos económicos

□ No confia en los medicos

1. ¿Cómo o dónde consigue el medicamento que toma?

□ Tienda □ Farmacia

□ Supermercado □ Ya lo tenía en casa

1. De una numeración del 1 al 10 (donde 1 significa Muy Malo y 10 significa Muy bueno) ¿Coloque en el espacio en blanco, el número que indica lo que piensa sobre la automedicación? _________

Además responda si la automedicación es:

□ Es buena para la salud.

□ Es buena para la economía.

□ Es buena ya que ayuda a los hospitales a no tener tanta gente.

□ No tiene ningún efecto.

□ No sabe qué efecto pueda tener.

□ Puede conducir a problemas de salud.

1. Al solicitar un medicamento sin receta de un doctor, ¿Usted se orienta con la ayuda de alguien?

□ Amigo/ Compañero

□ Dependiente de Farmacia

□ Familiar

□ Otro
